# Drug-Induced Cutaneous Reactions: A Clinically Oriented Review for Frontline Physicians

**DOI:** 10.7759/cureus.104176

**Published:** 2026-02-24

**Authors:** Ghaidaa S Elmehallawy, Reeman A Alharbi, Shaima Khaled A Kurdi

**Affiliations:** 1 College of Medicine, Taibah University, Madinah, SAU; 2 Dermatology, Madinah General Hospital, Madinah, SAU

**Keywords:** cutaneous adverse drug reactions, dermatology, dress syndrome, drug induced skin reactions, emergency medicine, stevens johnson syndrome, toxic epidermal necrolysis

## Abstract

Cutaneous adverse drug reactions are among the most frequently encountered adverse events in clinical practice and represent a significant source of morbidity and healthcare utilization. While many reactions are mild and self-limiting, delayed recognition of severe cutaneous adverse reactions can result in substantial morbidity and mortality. This review provides a clinically oriented overview of drug-induced cutaneous reactions with emphasis on early recognition, risk stratification, and management strategies relevant to frontline physicians. Drug-induced skin reactions encompass a broad clinical spectrum ranging from benign morbilliform eruptions to life-threatening conditions such as Stevens-Johnson syndrome, toxic epidermal necrolysis, and drug reaction with eosinophilia and systemic symptoms. Early identification, prompt discontinuation of the offending medication, and recognition of systemic involvement remain the most critical determinants of patient outcomes. A structured clinical approach based on temporal assessment, lesion morphology, and systemic evaluation can significantly improve outcomes and reduce progression to severe disease.

## Introduction and background

Cutaneous adverse drug reactions (CADRs) represent one of the most frequent manifestations of adverse drug events and remain a significant challenge in everyday clinical practice. Epidemiological estimates suggest that approximately 10-30% of adverse drug reactions involve the skin, with variability across outpatient dermatology settings and hospitalized populations [1,2]. Despite their frequency, early recognition remains inconsistent, particularly outside dermatology-focused environments, where initial clinical decisions often influence disease progression and overall patient outcomes.

The clinical importance of CADRs extends beyond dermatologic morbidity. Patients with suspected drug-related eruptions frequently present first to primary care physicians, internists, or emergency clinicians rather than dermatologists, emphasizing the need for structured clinical evaluation at the frontline of care. Delayed withdrawal of the offending medication is a well-recognized risk factor for progression to severe cutaneous adverse reactions (SCARs), including Stevens-Johnson syndrome (SJS), toxic epidermal necrolysis (TEN), and drug reaction with eosinophilia and systemic symptoms (DRESS) [2,3]. Mortality rates associated with TEN may exceed 30%, underscoring the necessity of early identification, appropriate risk stratification, and prompt therapeutic intervention during the initial clinical encounter [2].

Given the wide clinical spectrum and potential for rapid deterioration, clinicians must integrate careful history-taking, recognition of warning signs, and timely management strategies into routine practice. This review provides a clinically oriented overview of the pathophysiology, diagnostic approach, risk stratification, and management principles of drug-induced cutaneous reactions, with an emphasis on early recognition and multidisciplinary care.

Furthermore, contemporary studies highlight that delayed recognition of drug-induced eruptions remains a leading contributor to preventable morbidity, particularly in non-dermatology settings where early morphologic clues may be overlooked [4]. Advances in diagnostic frameworks highlight the importance of integrating medication timelines, systemic symptoms, and laboratory abnormalities to improve early risk stratification and clinical decision-making [5]. Emerging evidence also supports multidisciplinary management pathways and standardized assessment tools to optimize outcomes and reduce complications associated with severe cutaneous adverse reactions [6-8]. Recent literature has further refined diagnostic algorithms, pharmacogenomic screening strategies, and multidisciplinary management pathways for severe cutaneous adverse reactions, supporting a more structured frontline approach to early risk stratification [9-12].

This article is a clinically oriented narrative review intended to support frontline clinicians, including emergency medicine, internal medicine, and primary care physicians, in recognizing and managing cutaneous adverse drug reactions. Most drug eruptions are mild and self-limited; however, a minority represent severe cutaneous adverse reactions (SCARs) requiring urgent identification and intervention. The aim of this review is to synthesize clinically relevant evidence rather than perform a systematic review or quantitative meta-analysis. No meta-analysis, meta-regression, or formal quantitative synthesis was performed; findings are presented as a narrative clinical synthesis. The aim is to synthesize clinically relevant evidence rather than perform a systematic review or quantitative meta-analysis; therefore, findings are presented as a narrative clinical synthesis.

## Review

Approach to literature selection

This article was developed as a clinically oriented narrative review. Literature was identified through targeted searches of PubMed, MEDLINE, and Google Scholar, focusing on key terms including cutaneous adverse drug reactions, severe cutaneous adverse reactions, Stevens-Johnson syndrome, toxic epidermal necrolysis, DRESS syndrome, and drug hypersensitivity. Emphasis was placed on clinically relevant review articles, consensus guidelines, and high-quality observational studies published in English. Evidence was selected to support practical diagnostic and management principles for frontline clinicians rather than to perform a systematic review or quantitative synthesis. No formal risk-of-bias assessment or statistical pooling was conducted.

This narrative review was developed through targeted searches of PubMed/MEDLINE and Google Scholar, focusing on clinically relevant literature related to cutaneous adverse drug reactions and severe cutaneous adverse reactions. Priority was given to guideline statements, pharmacogenetic studies, consensus recommendations, and clinically oriented reviews. Emphasis was placed on publications addressing diagnostic frameworks, risk stratification, and management strategies relevant to frontline clinical settings. The goal was to provide a practical synthesis of representative evidence rather than an exhaustive systematic analysis.

Pathophysiology of drug-induced cutaneous reactions

Accurate recognition of drug-induced cutaneous reactions begins with understanding their epidemiology and clinical patterns, which vary widely depending on prescribing practices and patient characteristics across populations [1]. These reactions represent a heterogeneous group of inflammatory responses that may extend beyond isolated dermatologic findings to involve systemic organ dysfunction, with the skin often serving as an early clinical marker of broader immune activation [2].

Severe cutaneous adverse reactions arise from complex immunologic pathways, most commonly delayed type IV hypersensitivity reactions mediated by drug-specific T-cell activation. Cytotoxic immune responses directed at keratinocytes contribute to epidermal injury and explain the progression toward Stevens-Johnson syndrome and toxic epidermal necrolysis [3]. Epidemiologic studies further highlight the importance of host susceptibility and drug exposure patterns in determining clinical severity and outcomes [4].

Drug reactions may also involve immune-mediated organ damage beyond the skin, reflecting systemic inflammatory cascades that complicate diagnosis and management [5]. Mechanistically, reactive drug metabolites can function as haptens that bind host proteins and trigger cytokine-mediated inflammation, apoptosis, and epidermal detachment, underpinning many clinically significant reactions, including DRESS, SJS, and TEN [6]. In addition, non-immune mechanisms such as direct mast-cell activation, cumulative toxicity, and photosensitivity contribute to the diversity of clinical presentations [7]. Pharmacogenomic advances have demonstrated the role of genetic susceptibility, particularly the association between HLA-B*58:01 and severe allopurinol-induced reactions, supporting the growing role of personalized risk assessment [8].

Recent clinical literature further suggests that alterations in immune checkpoint signaling and cytokine network dysregulation may contribute to persistent inflammation and prolonged recovery in severe cutaneous adverse reactions [9]. Additionally, pharmacovigilance registries have demonstrated that early identification of high-risk medications and structured monitoring strategies significantly reduce progression to life-threatening reactions [10]. Emerging data also highlight the role of multidisciplinary clinical pathways and standardized severity scoring systems in improving prognostic assessment and guiding therapeutic escalation in complex presentations [11].

Clinical spectrum of drug-induced cutaneous reactions

The most frequently encountered CADR in clinical practice is the morbilliform, or exanthematous, drug eruption. These eruptions typically present as symmetrical erythematous macules and papules that begin on the trunk and spread to the extremities, usually developing one to two weeks after initiation of the offending medication. Antibiotics, anticonvulsants, and nonsteroidal anti-inflammatory drugs are among the most commonly implicated agents [1,6]. In the absence of systemic symptoms, morbilliform eruptions are generally benign and resolve following drug discontinuation and supportive therapy.

Urticarial drug reactions present with transient wheals and pruritus and may occur immediately or days after exposure. While many cases are mild, associated angioedema, particularly involving the lips, tongue, or upper airway, raises concern for progression to anaphylaxis and necessitates urgent evaluation. Fixed drug eruptions represent a distinctive pattern characterized by the recurrence of sharply demarcated erythematous or violaceous lesions at the same anatomical site upon re-exposure to the offending drug. These lesions often resolve with post-inflammatory hyperpigmentation and are commonly linked to antibiotics, NSAIDs, and antiepileptic drugs [6].

**Figure 1:**
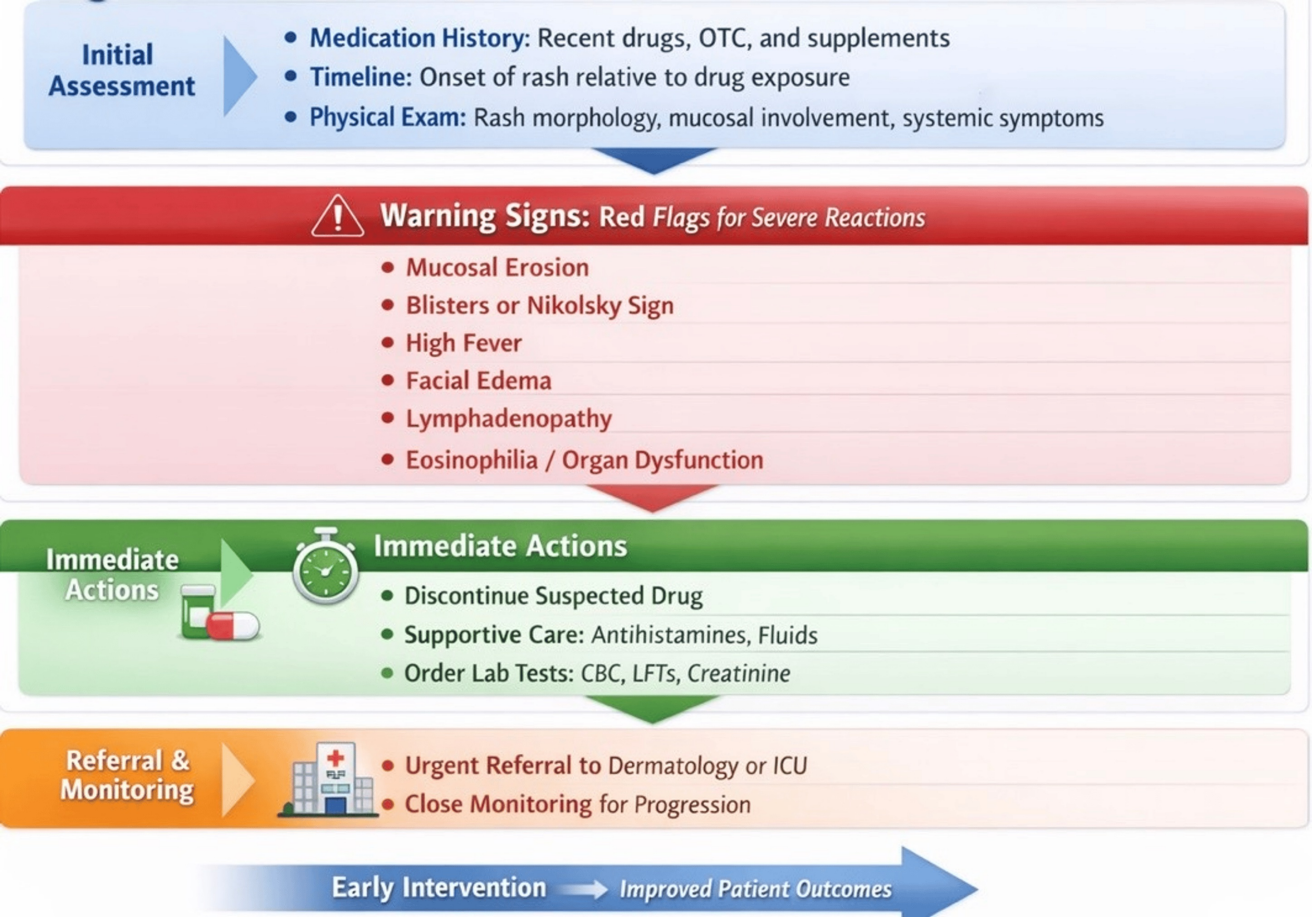
Clinical approach to suspected drug-induced cutaneous reactions Clinical algorithm outlining the stepwise evaluation and management of suspected drug-induced cutaneous reactions at the first point of care. The approach emphasizes detailed medication history, temporal correlation with drug exposure, identification of red-flag features suggestive of severe cutaneous adverse reactions, prompt discontinuation of the suspected offending drug, initiation of supportive care, and timely referral for specialist evaluation when indicated. Early recognition and intervention are critical to improving patient outcomes. Image Credit: Authors

Photosensitivity reactions are confined predominantly to sun-exposed areas and may manifest as phototoxic or photoallergic eruptions. Tetracyclines, thiazide diuretics, amiodarone, and certain retinoids are frequently implicated. Less commonly, drugs may induce acneiform or lichenoid eruptions, which can mimic idiopathic acne vulgaris or lichen planus and delay diagnosis when drug causality is not initially considered [7]. Recent observational studies emphasize that early differentiation between benign exanthematous eruptions and evolving severe cutaneous adverse reactions relies heavily on careful evaluation of lesion distribution, mucosal involvement, and systemic symptoms, which serve as early prognostic indicators [12]. Contemporary dermatology guidelines also highlight the importance of recognizing drug-specific latency patterns and high-risk medication classes, including anticonvulsants and allopurinol, to improve early diagnostic accuracy [13]. Furthermore, emerging registry-based analyses demonstrate that standardized clinical assessment tools and structured follow-up strategies enhance detection of progression from mild eruptions to severe disease states, supporting proactive monitoring in high-risk patients [14,15]. Key clinical features and diagnostic considerations are summarized within the narrative discussion below. The structured clinical approach to suspected drug-induced cutaneous reactions at the first point of care is illustrated in Figure 1.

Severe cutaneous adverse reactions

Severe cutaneous adverse reactions represent medical emergencies and demand prompt recognition and immediate intervention. Stevens-Johnson syndrome and toxic epidermal necrolysis are characterized by painful erythematous or purpuric macules, mucosal involvement, and epidermal detachment and exist along a disease spectrum differentiated primarily by the extent of body surface area involvement. Common causative agents include anticonvulsants, allopurinol, sulfonamides, and certain antibiotics [2,3].

Drug reaction with eosinophilia and systemic symptoms is a delayed hypersensitivity reaction that typically develops two to eight weeks after initiation of the offending drug. It is characterized by fever, widespread rash, facial edema, lymphadenopathy, eosinophilia, and internal organ involvement, most commonly affecting the liver and kidneys [3,4]. Acute generalized exanthematous pustulosis presents with a sudden onset of numerous sterile pustules accompanied by fever and neutrophilia and usually resolves rapidly after drug withdrawal.

**Figure 2:**
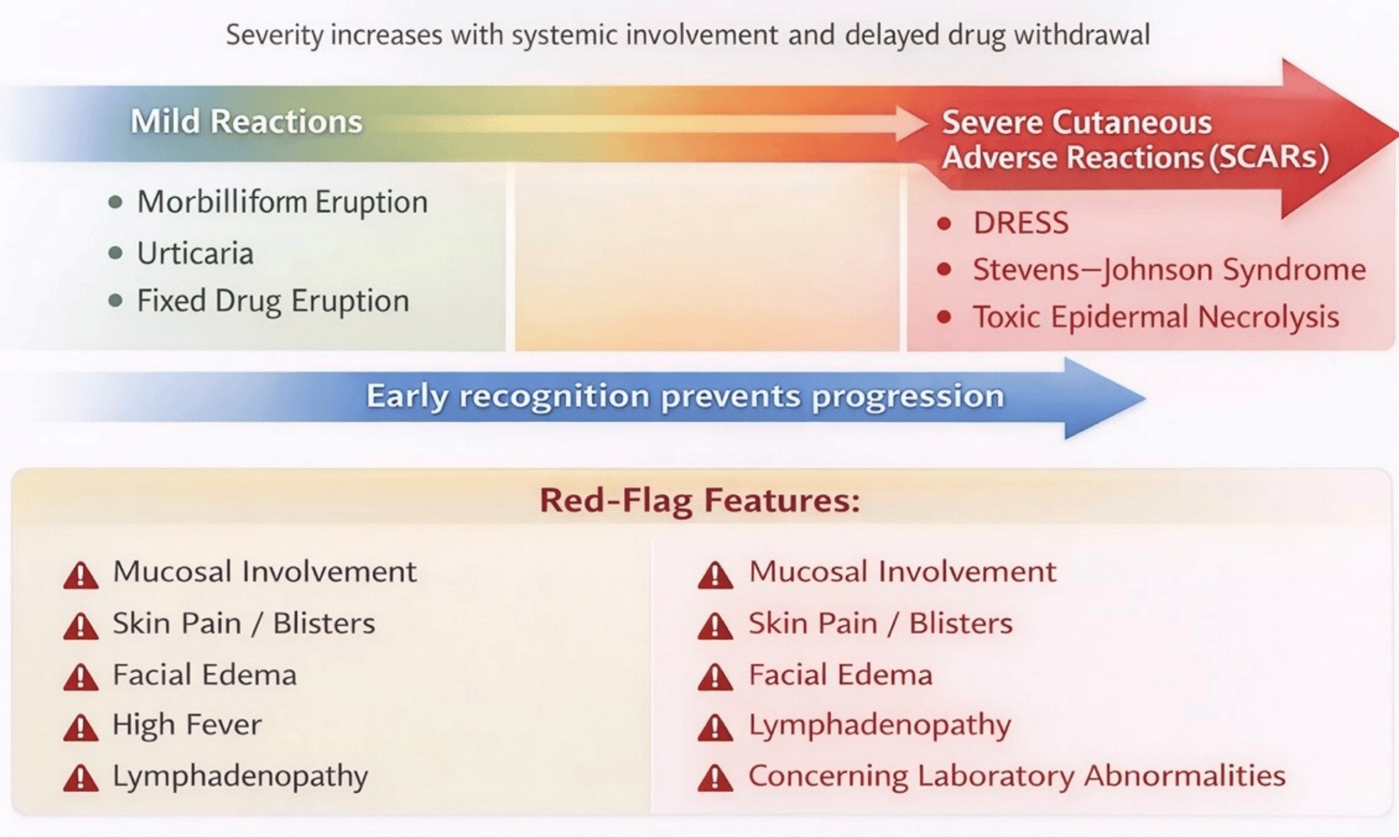
Spectrum of drug-induced cutaneous reactions and associated clinical severity Diagram illustrating the continuum of drug-induced cutaneous reactions from mild, self-limiting eruptions to severe cutaneous adverse reactions. Mild reactions include morbilliform eruptions, urticaria, and fixed drug eruptions, while severe reactions encompass drug reaction with eosinophilia and systemic symptoms, Stevens–Johnson syndrome, and toxic epidermal necrolysis. Progression along the spectrum is associated with increasing systemic involvement, higher morbidity and mortality risk, and delayed withdrawal of the offending medication. Early recognition of red-flag features and prompt intervention are essential to prevent disease progression. Image Credit: Authors

Across all SCARs, continued exposure to the causative drug is strongly associated with worse outcomes. Early recognition and immediate discontinuation of suspected medications are therefore the most important prognostic interventions [3,6]. The progression from mild exanthematous eruptions to severe cutaneous adverse reactions, together with associated red-flag features, is further illustrated in Figure 2.

Diagnostic approach in clinical practice

Diagnosis of CADRs relies primarily on clinical assessment. A thorough medication history, including recent prescription drugs, over-the-counter medications, and herbal supplements, is essential. Establishing a temporal relationship between drug exposure and rash onset is a critical diagnostic step. Physical examination should focus on lesion morphology, distribution, mucosal involvement, and signs of systemic illness such as fever or lymphadenopathy. Standardized clinical algorithms may improve diagnostic consistency across non-dermatology settings where early morphologic signs are frequently under-recognized. Laboratory investigations are not routinely required for mild reactions but are essential when systemic involvement is suspected. These typically include complete blood count, liver function tests, and renal function assessment. Skin biopsy may aid diagnosis in atypical or severe cases, but is generally performed after referral to dermatology or inpatient services [5].

Recent clinical literature emphasizes that integrating structured diagnostic algorithms and standardized severity assessment tools improves early differentiation between benign drug eruptions and evolving severe cutaneous adverse reactions [9]. Incorporating laboratory markers, temporal drug exposure patterns, and multidisciplinary evaluation pathways has been associated with improved diagnostic accuracy and earlier escalation of care in high-risk patients [10].

SCAR severity and causality assessment tools

Several structured tools assist clinicians in evaluating suspected severe cutaneous adverse reactions. SCORTEN is a validated severity scoring system primarily used in Stevens-Johnson syndrome and toxic epidermal necrolysis to estimate mortality risk and guide level-of-care decisions. ALDEN (Algorithm of Drug Causality in Epidermal Necrolysis) supports structured evaluation of drug causality based on timing, known drug risk, and alternative explanations. RegiSCAR criteria provide a standardized diagnostic classification of SCAR phenotypes, particularly DRESS syndrome, allowing improved consistency across studies and clinical practice. Incorporating these tools into frontline evaluation can enhance early recognition, risk stratification, and multidisciplinary coordination.

Management principles

The cornerstone of management of drug-induced cutaneous reactions is prompt identification and discontinuation of the suspected offending agent. In mild reactions, symptomatic treatment with topical corticosteroids and oral antihistamines is often sufficient, and symptoms typically resolve within days to weeks. Clear documentation of suspected drug allergy and patient education regarding future avoidance are essential components of care. Severe reactions require urgent referral and multidisciplinary management, often involving dermatology, internal medicine, and intensive care teams. Systemic therapies, including corticosteroids, immunosuppressive agents, or intravenous immunoglobulin, are initiated in-specialized settings based on disease severity and institutional protocols [3,6]. Emerging registry-based data further suggest that implementation of coordinated multidisciplinary care pathways and early supportive management strategies can reduce morbidity and optimize clinical outcomes in patients with severe drug-induced cutaneous reactions [11].

Special populations

Children pose diagnostic challenges due to the high prevalence of viral exanthems that mimic drug eruptions, necessitating cautious attribution of causality. Pregnant patients require careful consideration of drug safety and limited therapeutic options. Elderly patients are at increased risk due to polypharmacy and age-related changes in drug metabolism, warranting a lower threshold for investigation and referral [5].

Prevention and pharmacovigilance

Prevention of CADRs relies on rational prescribing, avoidance of unnecessary medications, and careful documentation of drug allergies. Patient education regarding early warning signs of severe reactions is particularly important. Reporting adverse drug reactions to national pharmacovigilance systems contributes to improved drug safety and population-level risk assessment. In selected high-risk populations, genetic screening for known HLA risk alleles may reduce the incidence of severe reactions, particularly with allopurinol [8].

Recent pharmacovigilance analyses emphasize the growing role of electronic health record surveillance systems and real-time adverse event reporting in identifying early safety signals and preventing recurrence of severe cutaneous reactions [12]. In addition, international consensus recommendations increasingly support pre-prescription risk stratification strategies, including pharmacogenetic screening and clinician education programs, to improve medication safety and reduce avoidable morbidity associated with high-risk drugs [13].

Limitations

This review is limited by its narrative design and reliance on previously published literature rather than a formal systematic review methodology. Variability in diagnostic criteria, reporting standards, and underreporting of adverse drug reactions may limit the generalizability of findings. Additionally, emerging pharmacogenetic data and evolving therapeutic strategies may not be fully captured.

Additionally, heterogeneity among published studies, including differences in diagnostic criteria, severity grading systems, and reporting methodologies, may introduce variability in outcome interpretation and limit direct comparison across cohorts [14]. Many available studies are retrospective or registry-based, which may lead to selection bias and incomplete capture of milder drug reactions managed in outpatient settings [15]. Future prospective multicenter investigations with standardized definitions and longitudinal follow-up are required to strengthen evidence quality and improve the generalizability of findings. Despite these limitations, current evidence provides valuable clinical guidance and highlights areas for future research aimed at improving early recognition and standardized management of drug-induced cutaneous reactions.

Recommendations

Frontline clinicians should maintain a high index of suspicion for drug-induced cutaneous reactions, particularly in patients with recent medication exposure and systemic symptoms. Early discontinuation of suspected offending agents, prompt risk stratification, and timely referral to dermatology or higher levels of care are essential to improving outcomes. Improved documentation and reporting of adverse drug reactions should be encouraged to strengthen pharmacovigilance efforts.

Implementation of structured clinical pathways, early severity scoring systems, and multidisciplinary collaboration between dermatology, internal medicine, and pharmacy teams may further enhance early recognition and reduce progression to severe cutaneous adverse reactions in high-risk patients [11,13].

Future directions

Future research should focus on standardized diagnostic algorithms, prospective studies evaluating early intervention strategies, and broader implementation of pharmacogenetic screening in high-risk populations. Integration of electronic health record-based decision support tools and artificial intelligence-assisted detection systems may further enhance early recognition and prevention of severe reactions. Emerging translational research focusing on immune biomarkers, pharmacogenomic screening, and real-world pharmacovigilance databases is expected to refine individualized risk stratification and guide precision-based therapeutic approaches in drug-induced cutaneous reactions [9,10,15].

## Conclusions

Drug-induced cutaneous reactions represent a frequent yet often underrecognized source of morbidity in clinical practice. Although many eruptions are mild and self-limiting, the major clinical challenge lies in distinguishing benign presentations from early manifestations of severe cutaneous adverse reactions, where delayed recognition and continued drug exposure may lead to significant morbidity and mortality. Recent advances in structured diagnostic algorithms, severity scoring systems, and multidisciplinary clinical pathways have improved early risk stratification and facilitated timely escalation of care.

As most patients initially present to frontline clinicians rather than dermatology specialists, early diagnostic decision-making remains critical. Integration of pharmacovigilance strategies, pharmacogenetic risk assessment, and electronic health record-supported surveillance systems may further enhance prevention and early detection of high-risk reactions. A coordinated clinical approach emphasizing temporal drug correlation, lesion morphology assessment, systemic evaluation, and prompt discontinuation of offending agents remains the cornerstone of management. Continued research focusing on translational biomarkers, individualized risk prediction, and standardized care pathways is essential to improving long-term outcomes and reducing preventable complications associated with drug-induced cutaneous adverse reactions.
